# When the first try fails: re-implementation of SIMPL in a general surgery residency

**DOI:** 10.1186/s12893-024-02557-2

**Published:** 2024-09-11

**Authors:** Phillip J. Hsu, Gregory Wnuk, Lisa Leininger, Samantha Peterson, David T. Hughes, Gurjit Sandhu, Jay B. Zwischenberger, Brian C. George, Staci Aubry

**Affiliations:** 1 Department of Surgery, University of Michigan, Ann Arbor, MI USA; 2 Department of Surgery, University of Kentucky, Lexington, KY USA

**Keywords:** Workplace-based assessment, Re-implementation, Evaluation, Barriers to usage, Change in culture

## Abstract

**Background:**

Workplace-based assessment (WBA) can facilitate evaluation of operative performance; however, implementation of WBA is sometimes unsuccessful. The American Board of Surgery Entrustable Professional Activities WBA project was launched in July 2023. Some programs will face the challenge of re-implementation of a WBA following previous failures. It is unknown what interventions are most effective for WBA re-implementation. Our goal is to identify barriers and facilitators to re-implementing SIMPL, an operative performance WBA.

**Methods:**

The System for Improving and Measuring Procedural Learning (SIMPL) was implemented at our residency in 2018, but usage rates were low. We interviewed residents and faculty to identify barriers to usage and opportunities for improvement. Residents reported that SIMPL usage declined because of several factors, including a low faculty response rate, while some faculty reported not responding because they were unable to login to the app and because usage was not mandated. We then re-implemented SIMPL using a plan based on Kotter’s Model of Change. To evaluate impact, we analyzed rates of SIMPL usage when it was first implemented, as well as before and after the date of re-implementation.

**Results:**

In September 2022, we re-implemented SIMPL at our program with measures addressing the identified barriers. We found that, in the six months after re-implementation, an average of 145.8 evaluations were submitted by residents per month, compared with 47 evaluations per month at the start of the original implementation and 5.8 evaluations per month just prior to re-implementation. Faculty completed 60.6% of evaluations and dictated feedback for 59.1% of these evaluations, compared with 69.1% at implementation (44% dictated) and 43% prior to re-implementation (53% dictated).

**Conclusions:**

After identifying barriers to implementation of a WBA, we re-implemented it with significantly higher usage by faculty and residents. Future opportunities exist to implement or re-implement assessment tools within general surgery programs. These opportunities may have a significant impact in the setting of national standardization of workplace-based assessment among general surgery residencies.

**Supplementary Information:**

The online version contains supplementary material available at 10.1186/s12893-024-02557-2.

## Introduction

Medical and surgical training programs across the world are increasingly utilizing competency-based resident education (CBRE) for evaluation. CBRE allows time-variable advancement, allowing outcomes or standards, rather than a rigid timeframe, to drive progression through training [1]. Inherent in CBRE is the utilization of workplace-based assessments (WBAs), which evaluate the clinical competence of trainees as they perform authentic, day-to-day patient care activities [2, 3]. WBAs are key to evaluating competency in Entrustable Professional Activities (EPAs), critical clinical tasks in which trainees are expected to work toward being able to perform without direct supervision [4]. Importantly, WBAs also allow trainees to receive feedback that is critical for improvement [5, 6].

Barriers to implementation of WBAs are widely reported across specialties, and sometimes lead WBAs to fail [7]. Commonly reported barriers include lack of engagement by trainees and/or assessors, time constraints inherently present during patient care, and the complexity of language during the assessment process. After failure of implementation, some programs consider re-implementing the WBA. However, it is unknown what interventions and approaches are most effective for re-implementation of WBAs.

In July 2023 the American Board of Surgery (ABS) launched the EPAs WBA project [8]. This initiative establishes a standardized WBA as an essential part of the evaluation of surgical residents at every general surgery residency in the United States. Thus, understanding how to best implement or re-implement a WBA is both important and timely, as many general surgery residency programs will need to overcome the barriers inherent in the implementation of a WBA.

One approach to overcoming institutional barriers to change is Kotter’s Model of Change, an 8-step model that systematically addresses the challenges inherent in creating lasting change [9]. It has been successfully utilized to effect change at academic medical centers, including in creating a culture of interprofessionalism at an institution with a previously “hierarchical” structure [10]. It has also been shown to be effective at the level of medical trainees, helping create and implement an online curriculum for addressing chronic pain [11]. We thus hypothesized that Kotter’s Model of Change could be used for reimplementing a WBA in a general surgery residency.

The ABS EPAs are implemented using an updated version of the Society for Improving Medical Professional Learning (SIMPL) assessment platform currently used at many general surgery residency programs nation-wide [12, 13]. Here, we aimed to identify barriers and facilitators to re-implementing the current SIMPL app at a general surgery program that had previously unsuccessfully attempted to implement its usage. We then document the re-implementation process, which utilized Kotter’s Model of Change, and analyze the effects of the re-implementation.

## Methods

### Data sources

We utilized operative performance data from SIMPL, a nonprofit research and educational quality improvement collaborative, collected through the SIMPL smartphone app (http://www.simpl.org, Boston, MA). We focused on data collected at a general surgery residency at a large Midwestern academic medical center. The app has been described in detail elsewhere [12, 13]. Briefly, within 72 h after a case, the resident or attending may initiate an assessment, prompting a notification on the mobile phone of the other member of the resident-attending dyad. In general, the resident initiates the assessment. The attending, in turn, completes 3 multiple-choice questions pertaining to resident intraoperative autonomy, resident intraoperative performance, and case complexity. The attending is also asked to dictate specific feedback to the resident. Importantly, assessments expire 72 h postoperatively, as research suggests that beyond this point assessments lose clarity [14].

### Setting

Usage of the SIMPL app was implemented at the described general surgery residency in 2018. However, enthusiasm among residents and faculty to adopt the app was limited, leading to low usage rates. Usage continued to decline to near zero between 2019–2022. In early 2022, the study team decided to help re-launch the app. We conducted informal interviews of senior residents who were present for the original implementation of SIMPL. Because our main goal was to encourage residents to provide candid responses that could create lasting change (rather than conduct a formal research study), we used informal interviews rather than standardized surveys. Through these conversations information was obtained about barriers to usage and opportunities for improvement. Faculty authors on the study team also spoke with their colleagues to better understand barriers to using SIMPL.

### Barriers to SIMPL usage

Through interviews with residents who had used SIMPL when it was first implemented, we identified two main factors leading to declining usage of the app. First, residents reported a low response rate from faculty, leading to a sense of frustration with the app. The app was seen as a poor use of time if faculty did not respond. It was also reported that reminders sent to faculty to complete the application could be perceived as bothersome. Second, some residents reported that the chief residents at the time of initial implementation did not find great utility in using SIMPL. This sentiment was reported to be passed down throughout the residency, contributing to low usage. While these challenges existed, residents also reported that SIMPL had some positive benefits. Several residents found that the dictated feedback was useful, and others appreciated that they could review old feedback and compare it to newer feedback.

Faculty authors spoke with colleagues and found two main barriers to usage. First, some faculty, especially ones who joined the institution after the original implementation, had never downloaded the app or set up their account. Second, utilization of the app was not enforced or mandated.

### Re-implementation

We then designed a plan for re-implementation utilizing Kotter’s Model of Change (Table 1) [9]. The Model has 8 steps: increase urgency, build guiding team, develop the vision, communicate for buy-in, empower action, create short term wins, don’t let up, and make change stick. Our urgency came in the form of the upcoming ABS Entrustable Professional Activities (EPAs) Project; because it would soon be mandatory to establish an effective WBA, program leadership was motivated to find a way to obtain resident and faculty usage of SIMPL. We built a guiding team by adding two members of SIMPL who could provide data about successful usage of the app nationwide as well as troubleshoot utilization of the app. We developed a vision that residents would log the majority of their cases using SIMPL, and communicated this vision with the residency’s Program Director and Administrative Chief Residents to gain buy-in. The Program Director then met directly with the Chair of Surgery as well as the Section Head of General Surgery, gaining further support.

**Table 1:** Kotter’s 8-step model of change and the steps taken by the study team to apply it to re-implementation of WBA

**Step**	**Action**
Increase urgency	Emphasized mandatory ABS EPAs Project and the need to establish an effective WBA
Build guidingteam	Recruited two key members of SIMPL to guide implementation based on previous successful experiences
Develop the vision	Established the goal that residents log the majority of their cases using SIMPL
Communicate for buy-in	Met with Residency Program Director and Administrative Chief Residents to gain buy-in
Empower action	Program Director led a grand rounds establishing the expectation that SIMPL be used for all cases
Creat short term wins	Presented increased response rates from faculty,encouraging further resident usage
Don't let up	Regularly displayed leaderboards of top users at grand rounds
Make changestick	Included SIMPL usage as an essential metric in semi-annual review of resident performance

With buy-in from program leadership, we empowered action through a short but tailored presentation at grand rounds by the Program Director in late 2022. This presentation outlined the expectation that SIMPL be used for all cases. Increased response rates from faculty created short term wins, encouraging further usage. Finally, we continued working toward our goal and made change stick by presenting brief monthly updates at grand rounds in the form of a leaderboard of top users for residents and faculty by name, thus promoting usage. Importantly, we also included SIMPL usage, defined as the number of SIMPL evaluations sent in a six-month period, as an essential metric in the semi-annual review of resident performance by the Program Director.

To address faculty concerns, we offered re-orientation for usage of the SIMPL app, and also sent faculty their login information via email. Although we considered making SIMPL utilization mandatory for faculty, we also considered the evaluation fatigue that is present for faculty. Instead, we relied on publicly sharing usage rates at faculty teaching conferences, which allowed faculty to recognize that the app was being used by their colleagues and thus encourage their usage.

### Analysis

To evaluate the impact of our intervention, we analyzed rates of SIMPL usage by residents and faculty at three timepoints: when it was first implemented in 2018; just prior to re-implementation in late 2022; and after re-implementation. We defined “regular responders” as faculty who completed 50% or more of the evaluations sent to them by trainees. We also analyzed faculty demographics of rank and gender to identify whether there was any difference in their response rate after re-implementation All analyses were performed using Microsoft Excel, and images were created using Graphpad Prism version 7.0b (Boston, MA, USA).

## Results

### Effect of re-implementation

**Fig. 1:**
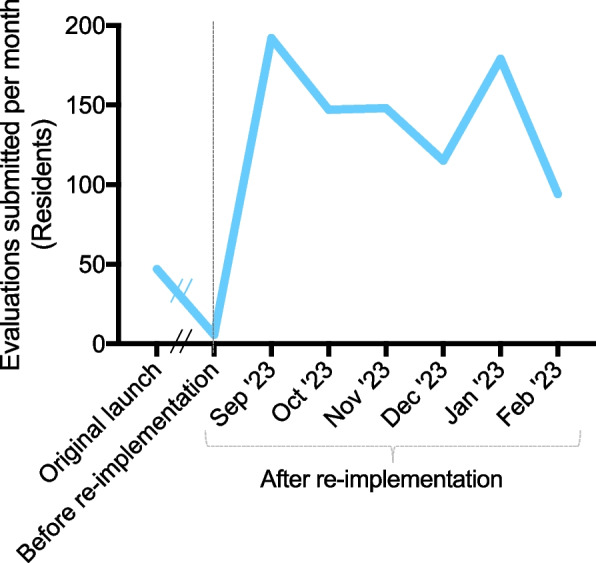
Number of evaluations submitted by residents per month at original launch of SIMPL, immediately before re-implementation, and in the six months after re-implementation

We re-implemented SIMPL at the described general surgery residency program in September 2022 with a strong endorsement from the Program Director, faculty, and Administrative Chief Residents. In the six months after re-implementation, an average of 145.8 ± 37.1 evaluations were submitted by residents each month (Fig. 1). This was significantly greater than the number evaluations submitted per month at the start of the original implementation (47 ± 7.6, *p* = 0.002). Of note, the number of evaluations submitted per month just prior to re-implementation had dropped to 5.8 ± 3.8 (*p* < 0.001). Regression analysis of the number of evaluations submitted in the first six months after re-implementation did not show a statistically significant change over time (95% CI [-33.9, 9.5]).

**Fig. 2:**
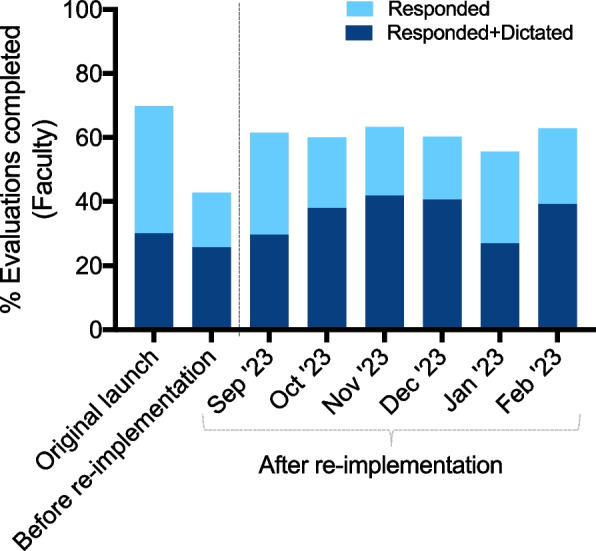
Proprotion of evaluations submitted by residents that were completed by faculty at original launch of SIMPL, immediately before re-implementation, and in the six months after re-implementation. Proportion of faculty responses that include dictations are indicated in dark blue

A large component of re-implementation was to promote completion of evaluations by faculty and to encourage faculty to dictate specific feedback in their responses. We thus analyzed the response rate of faculty before and after re-implementation. In the six months after re-implementation, faculty completed 60.6% of evaluations, compared with 69.1% of evaluations completed at implementation and 43% of evaluations prior to re-implementation (Fig. 2). After re-implementation, faculty dictated feedback in 59% of the evaluations they completed, compared with 33% of the time at implementation and 44% of the time just prior to re-implementation.

Because residents reported that one of the largest drivers of resident usage of SIMPL was the response rate from faculty, we analyzed whether any faculty demographics affected their response rate after re-implementation. We first analyzed whether academic rank affected response rate. We found that 50% of assistant professors (12 of 24), 45% of associate professors (10 of 22), and 50% of professors (13 of 26) were regular responders who completed more than 50% of the evaluations sent to them (Supplemental Fig. 1A). When analyzing whether faculty gender affected response rate, we found that 48% of female faculty (13 of 27) were regular responders, similar to the 49% of male faculty (22 of 45) who were regular responders (Supplemental Fig. 1B). Thus, neither academic rank nor gender affected the response rate of faculty.

We also analyzed whether post-graduate year (PGY) or resident gender affected the number of evaluations residents sent to faculty. We found that there were large variations in usage among residents of all classes (between 0 and 19 evaluations submitted per month), and that there were no significant differences in the number of evaluations sent between PGY years. Similarly, there were no significant differences in the number of evaluations sent between female and male residents.

## Discussion

Here, we have reported our experience re-implementing a widely used WBA. We approached the process using a systemic method, adopting Kotter’s Model of Change. After re-implementation, usage by faculty and residents increased significantly. However, there were still gaps in usage, including in the response rate by faculty.

We found that several challenges were faced during the initial implementation of SIMPL, generally centering around the lack of a culture of usage by both residents and faculty. Some residents did not feel that it would be useful, and some faculty either did not have access to the app or did not see it as necessary. Several residents reported that decreased usage led to increased dissatisfaction with the app, ultimately further decreasing usage. These lessons thus informed the re-implementation strategy to center around the expectation of usage. By publicly sharing usage rates of individual residents and faculty at teaching conferences, we worked to create a culture where SIMPL became an important part of every case.

We believe that the increase in usage by residents was motivated by several factors. Because the Program Director established SIMPL usage as a program requirement, residents felt personal responsibility to utilize the app. Moreover, the expectation to use SIMPL decreased resident concerns that sending evaluations to faculty would be seen as bothersome. In turn, the increased response rate by faculty likely also led to increased willingness of residents to utilize the app. These observations are consistent with prior literature exploring motivations for using the SIMPL WBA [15, 16].

Another factor that motivated resident usage was the quality and timeliness of the feedback received. During informal conversations with residents after re-implementation, there was a common theme that having feedback for a specific case made the feedback more actionable – that residents could “pinpoint the movement or thought process” referred to by the attending. Additionally, some residents felt that having immediate, real-time feedback allowed them to progress more quickly when performing multiple cases with the same attending.

Despite the significant increase in usage, implementation challenges still remain. Many, but not all, residents use SIMPL reliably. There was wide variation in usage, with some residents not having used SIMPL at all despite being told that it was a program requirement. This variation was present in all PGY years. While further study will be necessary to better elucidate remaining barriers to successful re-implementation, informal conversations with residents did provide some initial insights. One junior resident felt that, because they were being evaluated on operations that may have been repetitive or less complex, the feedback was often vague (e.g. “nice job with a new procedure”). Other residents expressed the continued frustration that faculty do not always respond. Indeed, while faculty response rate and dictation rate have both improved, only about 50% of faculty are “regular responders,” completing 50% or more of the evaluations they were sent. The program is taking measures to encourage further faculty involvement, including reaching out to faculty who repeatedly do not complete evaluations. However, more work will be required to improve both resident and faculty usage.

By sharing our own journey in implementing WBA, we report successful strategies toward the wider implementation of the ABS EPAs which launched this year. We suspect many programs will face challenges gaining buy-in from faculty and residents. The mandate from the ABS should drive participation. For those programs that are not able to successfully implement EPAs (or sustain implementation, as in our case), we found that a systematic approach to reimplementation is particularly valuable. Re-implementation is very time consuming, so the best strategy is to avoid re-implementation by maintaining momentum from the very beginning. The strategies discussed above may be used for both implementation and re-implementation.

An important point of discussion is how to achieve long-term retention and usage when morale fades. We believe there are lessons that can be learned from previous usage of the model in other fields. One important finding has been that having a leader who truly advocates for the change, rather than paying “lip service,” is crucial to the continued retention of the change [9]. In the case of a general surgery residency, having a Program Director keep the WBA as an essential part of each resident’s evaluation could be a way to implement this. Another important finding has been that leaders of successful efforts use the results of the change to address even bigger problems. By using the change as a foundation to engineer projects that are larger in scope, the change becomes an essential component of the organization’s culture. In the case of a general surgery residency, the WBA can be utiliized as an essential component of helping grant increased autonomy, a goal shared by both resients and faculty.

This study has limitations. Firstly, it represents the experience of one general surgery residency, and thus may not be generalizable to other residencies. As the general surgery residency is based at a tertiary care academic medical center, our experience may be different from those of community programs. Secondly, our work describes the results of the first six months of re-implementation, so it is too early to conclude whether re-implementation will create lasting success. Third, given the small sample size and wide variation in usage, it is difficult to truly tell whether factors such as academic rank, PGY year, or gender affect SIMPL usage.

## Conclusions

We identified and addressed barriers to implementation of a widely used WBA, then re-implemented it in our general surgery residency. We found main barriers included a cycle of low response rates leading to decreased usage of the app, and that our re-implementation strategy led to significantly higher usage by faculty and residents. As mandating a WPA does not necessarily guarantee usage, residents and faculty must perceive value to continue utilization. Although usage increased, a proportion of residents and faculty still have not adopted regular WBA usage. As WBA usage is becoming standardized nationally among general surgery programs, it will be important to understand whether programs are successful at re-implementing WBAs and how. Further data about how best to re-implement WBAs will be forthcoming; future studies on strategies for successful re-implementation will be impactful.

## Supplementary Information

Supplementary Material 1: Supplemental Fig. 1. (A) Academic Rank and (B) Gender of faculty do not affect response rate.

## Data Availability

Data consists of SIMPL usage data by residents at our institution as well as interview data. All data is available upon reasonable request to Phillip J. Hsu (phillipjhsu@gmail.com).
