# Supplementary figures and images for: When the first try fails: re-implementation of SIMPL in a general surgery residency
Source: BMC Surg. 2024 Sep 11;24:257. doi: 10.1186/s12893-024-02557-2 (PMC11389305; doi:10.1186/s12893-024-02557-2)

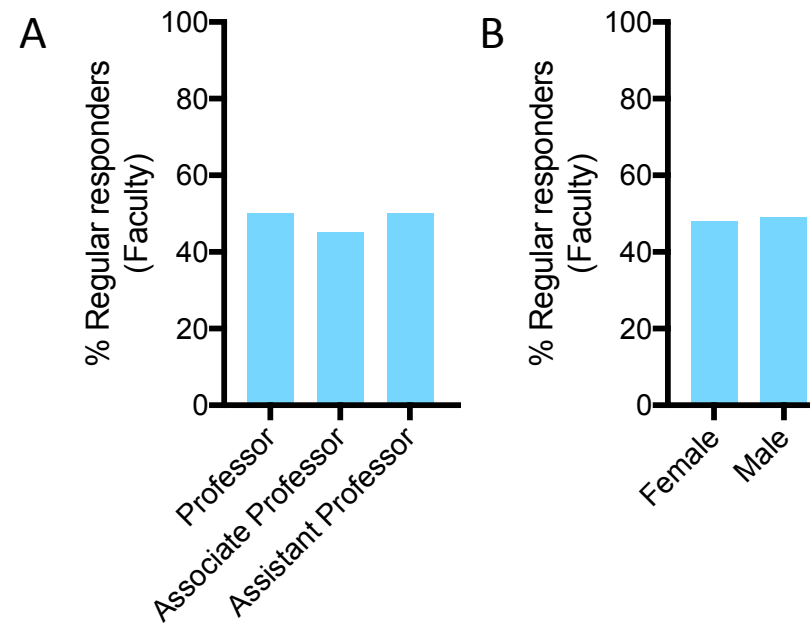

Supplemental Figure 1

Supplement: Supplementary file 1 — Supplementary Material 1: Supplemental Fig. 1. (A) Academic Rank and (B) Gender of faculty do not affect response rate. [file 12893_2024_2557_MOESM1_ESM.pdf]
